# Three-dimensional simulation for fast forward flight of a calliope hummingbird

**DOI:** 10.1098/rsos.160230

**Published:** 2016-06-08

**Authors:** Jialei Song, Bret W. Tobalske, Donald R. Powers, Tyson L. Hedrick, Haoxiang Luo

**Affiliations:** 1 Department of Mechanical Engineering, Vanderbilt University, Nashville, TN 37235, USA; 2 Field Research Station at Fort Missoula, Division of Biological Sciences, University of Montana, Missoula, MT 59812, USA; 3 Department of Biology, George Fox University, Edwards-Holman Science Center, Newberg, OR 97132, USA; 4 Department of Biology, University of North Carolina at Chapel Hill, Chapel Hill, NC 27599, USA

**Keywords:** flapping wings, hummingbirds, forward flight, unsteady aerodynamics

## Abstract

We present a computational study of flapping-wing aerodynamics of a calliope hummingbird (*Selasphorus calliope*) during fast forward flight. Three-dimensional wing kinematics were incorporated into the model by extracting time-dependent wing position from high-speed videos of the bird flying in a wind tunnel at 8.3 m s^−1^. The advance ratio, i.e. the ratio between flight speed and average wingtip speed, is around one. An immersed-boundary method was used to simulate flow around the wings and bird body. The result shows that both downstroke and upstroke in a wingbeat cycle produce significant thrust for the bird to overcome drag on the body, and such thrust production comes at price of negative lift induced during upstroke. This feature might be shared with bats, while being distinct from insects and other birds, including closely related swifts.

## 1. Introduction

Hummingbirds are distinguished and extremely agile flyers among birds. They are capable of not only sustained hovering flight, but also fast forward flight and various rapid manoeuvers. Recent studies of fluid dynamics have mainly focused on hummingbirds’ unique hovering capability and unsteady aerodynamics associated with the wings that move in a relatively horizontal plane [1–6]. These studies include both experimental measurement using digital particle image velocimetry (PIV) [1–4] and computational fluid dynamics simulations [5,6]. Despite having a vertebrate musculoskeletal configuration, hummingbirds in hovering move their wings forward during downstroke and backward with an inverted angle of attack during upstroke, i.e. a flight strategy that is used by many insects (e.g. fruit flies and bumblebees) as well as bats. As a result, both downstroke and upstroke of the wings generate weight support, even though it has been shown for some of the hummingbirds that downstroke produces approximately twice as much as weight support as upstroke [2,5]. Closely associated with the translation and pitching motions of the wings, the unsteady flow around hummingbirds has similar features as those of the insect wings [7–10]. For example, the flow is largely separated from the top wing surface; leading-edge vortices are formed near the wing surface during both downstroke and upstroke, and they play an important role in the force production. From a biological perspective, such similarity with insects appears to be owing, in part, to evolved specialization for high transmission ratio (i.e. the ratio of contractile velocity of the muscle to tangential velocity of the wingtip) in the hummingbird wing [11].

Compared with hovering flight, the forward flight of hummingbirds and its fluid dynamics have been much less studied. Tobalske *et al.* [12] performed comprehensive measurement of the flight kinematics of the rufous hummingbirds in a wind tunnel at speeds ranging from zero (hovering) to 12 m s^−1^. The data they obtained include the body orientation angle, wingbeat frequency, wingbeat amplitude, stroke plane angle, wingtip trajectory and time-dependent variables such as the wing chord angle and wing area. In general, as the flight speed increases, the birds align their bodies more parallel to the flow to reduce drag, and the stroke plane becomes more vertical, which is beneficial for thrust production. Based on their data, the advance ratio, *J*, defined as the ratio between the flight speed, *U*, and the average wingtip speed, *U*_*tip*_, is between *J*=0 for hovering and *J*=1.5 for the maximum speed at 12 m s^−1^. In comparison, insects typically have an advance ratio of *J*<1 [13,14]. For example, fruit flies and bumblebees have the advance ratio at 0.25 and 0.6, respectively [15,14]; so their wing speed is much faster than the flight speed. Such a difference in the flight dynamics implies that there should exist a significantly different force production mechanism in the hummingbird wings than that of those insect wings during forward flight.

Among animal flyers, there are several types of thrust production mechanisms for the forward flight mode. One common type is the so-called ‘backward flick’ [16], which is used by many insects, e.g. bumblebees [14,15,17], also by bats [18] and birds that have relatively pointed wings (e.g. doves) [19] during slow flight with *J*<1. In this type, the stroke plane tilts forward and the backward speed of the wing during upstroke is faster than the flight speed. Thus, the aerodynamic lift generated by the wings is directed forward during upstroke and functions as thrust. Another type is the paddling mode found in a recent study of fruit flies at speed of 0.43 m s^−1^ [20]. The advance ratio of fruit flies is near *J*=0.25 [14]. In this case, the stroke plane of the insects remains nearly horizontal, and the angle of attack of the wings at upstroke is much greater than that at downstroke. Thus, large drag is produced in the forward direction as a drag-based thrust mechanism. For large birds at cruising flight, the advance ratio is usually above one [21], and thrust is typically generated during downstroke when the leading-edge tilts downward to redirect the aerodynamic lift forward for simultaneous weight support and thrust production [22]; during upstroke, birds with rounded wings tend to fully flex and feather their wings, thus producing little force [22–25], but those with pointed wings such as pigeons, doves, cockatiels and parakeets sweep their wings on upstroke in fast flight and continue to produce weight support, albeit at a reduced level compared with downstroke [26,27].

Since the advance ratio of hummingbirds varies from zero to above one, it is possible that they use disparate force production mechanisms at different flight speeds. From Tobalske *et al.* [12], the tip trajectory of the hummingbirds at slow flight speeds is highly skewed, when viewed from a global coordinate system, and resembles that of insects. Thus, the backward flick mode is probably employed for thrust production. However, at fast speeds, it is not clear whether the hummingbirds become more like other birds, or if they adopt a different flight strategy. To answer this question, it is necessary to examine the detailed wing motion at those speeds. In addition to the kinematic analysis, accurate calculation of the forces is needed, as the flow under consideration is highly three-dimensional and involves unsteady effects beyond limitations of quasi-steady models.

In the current study, we aim to understand lift and thrust production of hummingbirds during fast forward flight. The flow field and behaviour of vortices will be investigated along with aerodynamic forces. In addition, we will compare force generation mechanisms between hummingbirds and insects, other birds including the closest relatives, swifts, as well as bats. Following the approach in a previous study of hovering flight of the hummingbird [5], we develop a high-fidelity computational model that incorporates the realistic kinematics of the bird wings and adopt three-dimensional numerical simulations to resolve the unsteady flow.

## 2. Model configuration and simulation approach

### 2.1 Reconstruction of the wing kinematics

**Figure 1.**
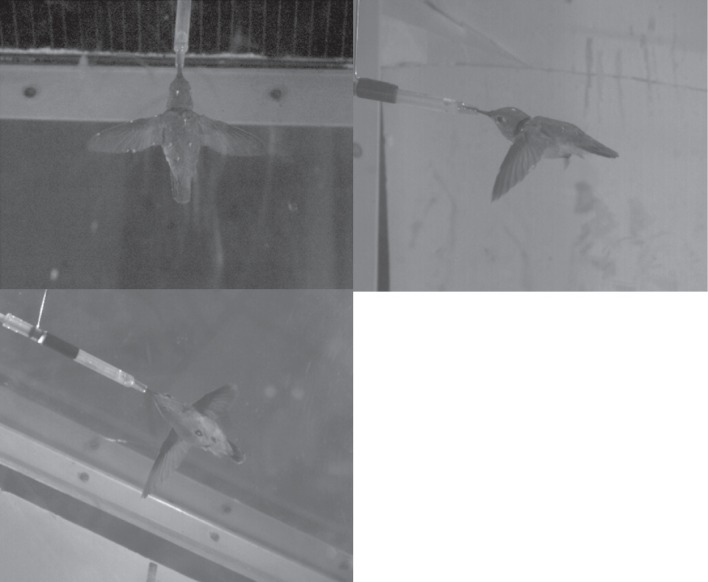
Camera views of the hummingbird flying in the wind tunnel.

A female calliope hummingbird (*Selasphorus calliope*) was the study subject, whose basic morphological data are provided in table 1. The experimental study was conducted to obtain the wing kinematics at a sustained flight speed of *U*=8.3 m s^−1^, at which the wingbeat frequency is 45.5 Hz while the stroke plane angle between the stroke plane and the horizontal is 67.9°. In the experiment, the bird was placed in an open-circuit, variable-speed wind tunnel with a feeder at the middle of the tunnel, and it was trained to adapt to the wind while feeding. We recorded flight kinematics of the hummingbird using three high-speed cameras distributed outside the wind tunnel: 1 Photron SA-3 (Photron USA Inc., San Diego, CA, USA) and 2 two Photron 1024 PCI, all with electronically synchronized shutter timing. Two cameras were placed dorsally to the bird, and one was placed laterally, as shown by the views in figure 1. Video recordings were made at 1000 Hz with a shutter speed of 1/10 000 s. The working section of the tunnel is 85 cm in length, square in cross section, 60×60 *cm*^2^ at the inlet and increasing to 61.5×61.5 *cm*^2^ at the outlet to accommodate boundary-layer thickening [28]. Maximum deviations in velocity within a cross section are less than 10% of the mean; the boundary layer is less than 1 cm thick and turbulence is 1.2%.

**Table 1.** Morphological and basic kinematics data of the hummingbird used in the study.

parameter	value
mass	*M*=2.8 g
stroke plane angle	*β*=67.9°
stroke amplitude	*Φ*=102.5°
wingbeat frequency	*f*=45.5 Hz
wing length	*R*=4.51 cm
single wing area	*S*=5.18 cm^2^

**Figure 2.**
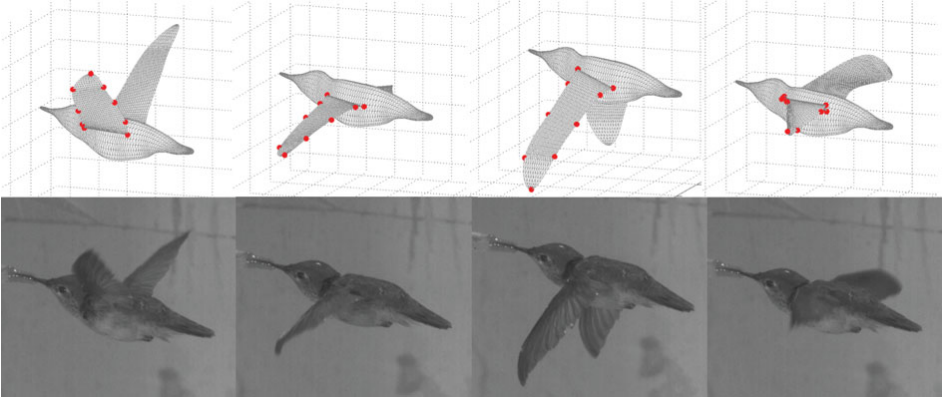
Reconstructed wing positions and corresponding snapshots from the camera view.

**Figure 3.**
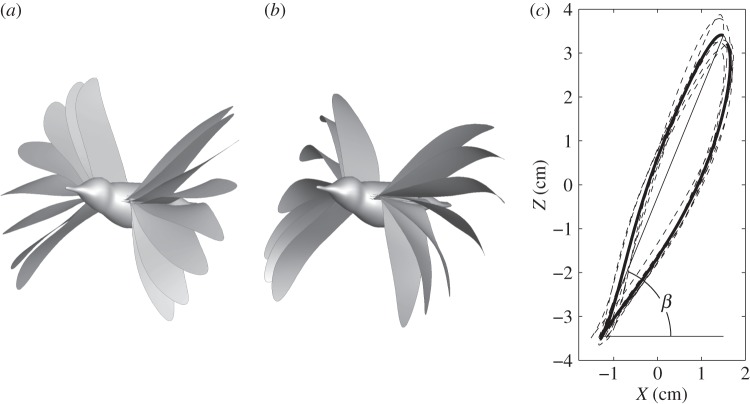
Reconstructed wing position of the hummingbird within one beat cycle: (*a*) downstroke, (*b*) upstroke and (*c*) the left wingtip trajectory as viewed in a body-fixed coordinate system, where the thick line is the phase-averaged and the thin dashed line is the instantaneous data.

After the videos were taken, a custom Matlab program [29] was used to track the markers frame by frame and to extract their three-dimensional coordinates. These markers were pre-labelled on the wings using non-toxic paint, and they included five points on the leading edge, on at the wingtip and three on the trailing edge, as shown in figure 2, where a comparison of the reconstructed model with the camera view shows that the instantaneous wing position and deformation are well captured. In a similar study for the hovering hummingbird [5], a principal components analysis verifies that these points are sufficient to characterize the wing motion. The wing geometry reconstruction process is similar to that in the previous study [5]. The wing profile was constructed using spline interpolation through the marker points, and the wing surface was then built using triangular elements within the profile. The bird body was reconstructed using the camera views of the hummingbird. In the current reconstruction, a single wing consists of 1335 elements and 718 nodes, while the body surface consists of 3560 elements and 1782 nodes. A total of 13 cycles of wingbeats during steady flight were captured, and each cycle contains approximated 22 frames. To increase the time resolution of the wing position, the trajectories of the wing mesh nodes are also refined by spline interpolation in time. Seven cycles of wingbeats were reconstructed from the imaging data and used for the simulation. Figure 3 shows a sequence of wing positions within a cycle and also the wingtip trajectory (see the electronic supplementary material for an animation).

### 2.2 Wing kinematics

**Figure 4.**
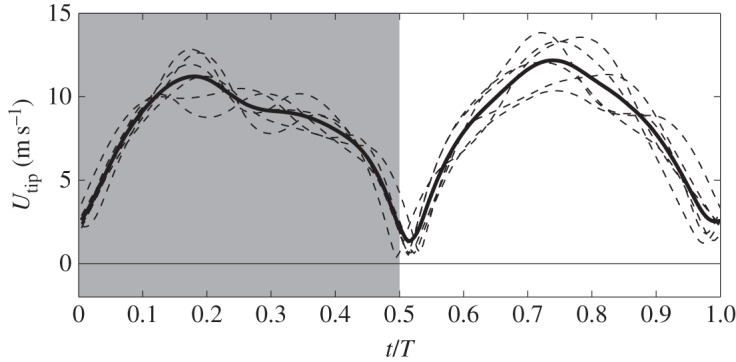
Instantaneous (thin dashed lines) and average (thick line) wingtip velocity. In all figures, grey background represents downstroke and white represents upstroke.

 Figure 4 shows both the instantaneous and phase-averaged wingtip velocity of the hummingbird. The upstroke has slightly higher velocity than downstroke. The peak value is at 11.22 m s^−1^ for downstroke and 12.18 m s^−1^ for upstroke. The wingtip velocity averaged throughout the cycles is 8.14 m s^−1^, which gives the advance ratio *J*=1.02. The wing area can be calculated from the reconstructed kinematics and it varies between 5.34 cm^2^ during downstroke and 5.03 cm^2^ during upstroke, with average at *S*=5.18 cm^2^.

**Figure 5.**
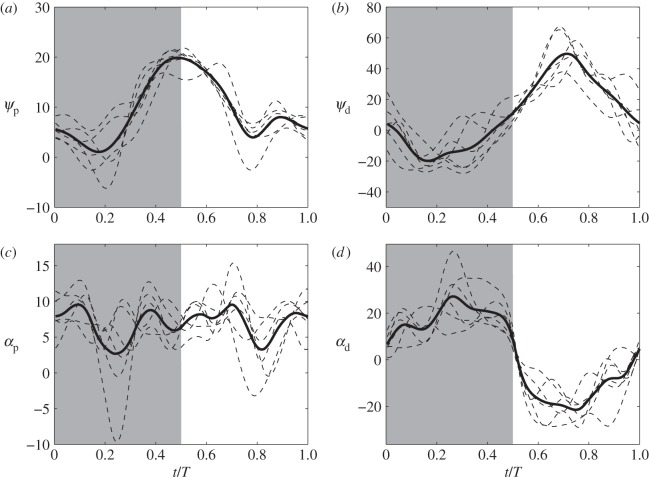
Chord angle *ψ* (*a*,*b*) and effective angle of attack *α* (*c*,*d*) for a proximal chord at $\hat{r}=0.15$ (*a*,*c*) and a distal chord at $\hat{r}=0.90$ (*b*,*d*).

From figure 3, wing twist along the axis and spanwise bending in a cycle are evident. To characterize the position of a cross section of the wings, we define the chord angle *ψ* as the instantaneous angle between the chord and the flight direction. The angle of attack, *α*, is defined as the angle between the chord and the relative flow direction that combines both the freestream velocity and the translational velocity of the chord at the leading edge. These two angles are plotted in figure 5 for two chords and five cycles, one proximal chord at dimensionless location $\hat{r}=r/R=0.15$ and one distal chord at $\hat{r}=0.9$, which are denoted by subscripts p and d, respectively.

**Figure 6.**
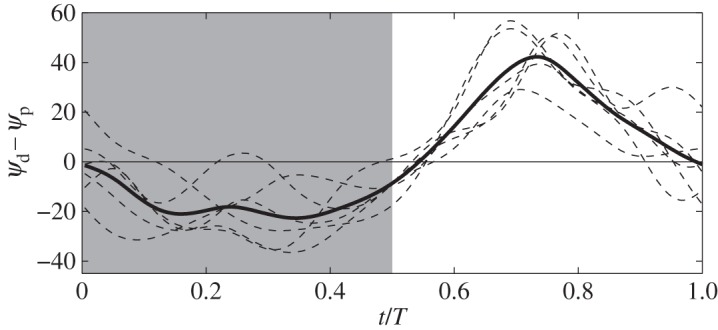
Wing twist as measured using the difference between two chord angles.

From these plots, we can see large differences between the proximal chord and distal chord. For the proximal chord, the chord angle *ψ*_*p*_ and angle of attack *α*_*p*_ are both positive during the entire cycle. For the distal chord, these angles change the sign and vary significantly. During the downstroke, *ψ*_*d*_ is negative, i.e. the leading edge tilting downward, but *α*_*d*_ is positive owing to fast translation of the chord. During upstroke, *ψ*_*d*_ is positive, i.e. the leading edge tilting upward, but the *α*_*d*_ is negative, indicating that the pressure surface and the suction surface are swapped at that moment. Wing twist can be described by the difference between the two chord angles, *ψ*_*d*_−*ψ*_*p*_, which is plotted in figure 6. It is shown that the twist angle reaches its extreme value during mid-downstroke and mid-upstroke; however, it is more pronounced during upstroke (near 40°) than during downstroke (near 25°). These differences between the proximal section and the distal section lead to highly non-uniform pressure distribution on the wing surface as shown later.

### 2.3 Simulation set-up and verification

**Figure 7.**
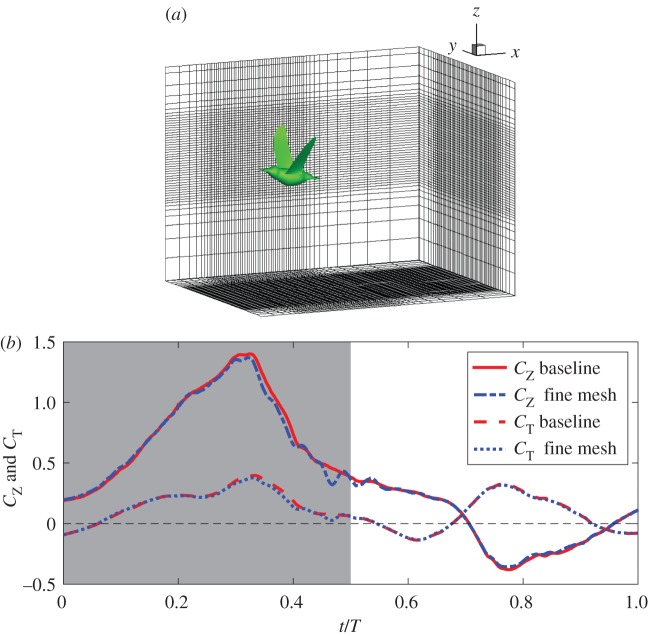
(*a*) Baseline mesh around the bird (only one out of every 12 points in each direction is shown). (*b*) Force comparison between baseline simulation and fine mesh simulation.

In the model, the Reynolds number, defined as $U\bar{c}/\nu$, is set to be *Re*=3000, where $\bar{c}$ is the average chord length and *ν* is the kinematic viscosity. The flow is assumed to be governed by the viscous incompressible Navier–Stokes equation, which is solved by an in-house code that adopts a second-order immersed-boundary finite-difference method. The code is able to handle large displacement of the moving boundaries [30]. A fixed, non-uniform, single-block Cartesian grid is employed to discretize the domain (figure 7*a*). The rectangular domain is 25×20×16 cm^3^. For the simulation, 704×842×560 (333 million) points are used for the baseline simulation. A finer mesh is also used in the simulation to verify grid convergence. Both of these meshes have maximum resolution around the wing, which is $\frac{1}{60}\;\mathrm{cm}$ in all three directions for the baseline case and $\frac{1}{70}\;\mathrm{cm}$ for the refined case. The simulation was run in parallel using domain decomposition and Message Passing Interface (MPI). The time step is Δ*t*=5 μ*s*, which leads to approximately 4400 steps per wingbeat cycle. A multigrid method was employed to accelerate convergence of the Poisson solver. A total of 96 processor cores were used for the baseline case, and 128 cores for the refined case.

The vertical force *F*_*Z*_ and thrust *F*_*T*_=−*F*_*X*_ generated by one wing are normalized by the fluid density, *ρ*, the flight speed, *U*, and the surface area of the wing according to

2.1 $$C_{Z}=\frac{F_{Z}}{(1/2)\rho U^{2}S}\;\mathrm{and}\;C_{T}=\frac{F_{T}}{(1/2)\rho U^{2}S}.$$

The lift and drag on the bird body, *F*_*Z*_*b*__ and *F*_*D*_*b*__, are normalized in the same manner. The aerodynamic power coefficient of one wing is defined as

2.2 $$C_{P}=\frac{\int f\cdot u\;dA}{(1/2)\rho U^{3}S}.$$

where f is the stress on the wing surface, and u is the velocity of a point on the wing in the body-fixed coordinate system.

The simulation results for two wingbeat cycles from both meshes are shown in figure 7 and table 2 for comparison. In figure 7 and also other figures from herein, the shaded area indicates downstroke, while the white area indicates upstroke. These results include the time-averaged lift and thrust of one wing and also lift and drag of the bird body. From the table, we see that the maximum difference of all the forces is less than 5%. Thus, the baseline resolution is deemed satisfactory for the current study.

**Table 2.** Comparison of the force coefficients for the wings and body from the two different meshes, where *C*_*Z*_ and *C*_*T*_ are for the vertical force and thrust of one wing, respectively, and *C*_*Z*,b_ and *C*_*D*,b_ are for the vertical force and drag of the body, respectively.

	*C*_*Z*_	*C*_*T*_	*C*_*Z*,b_	*C*_*D*,b_
baseline	0.374	0.112	0.239	0.151
fine mesh	0.381	0.117	0.238	0.150
difference (%)	1.84	4.27	0.42	0.67

## 3. Results and discussion

### 3.1 Aerodynamic forces

**Figure 8.**
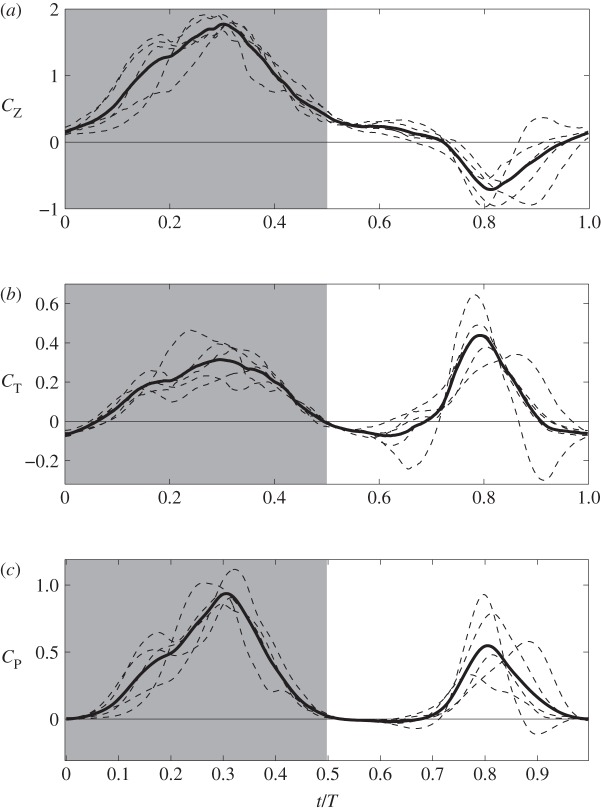
Force production and aerodynamic power consumption of each hummingbird wing: (*a*) vertical force coefficient, (*b*) thrust coefficient and (*c*) power coefficient. In each case, the thin dashed lines are the instantaneous data, and the thick line is the phase-averaged data.

The force and power coefficients are shown in figure 8, which include both instantaneous and phase-averaged data. The cycle-averaged data are listed in table 3 for an entire cycle and for downstroke/upstroke separately. Figure 8*a* shows that the weight support is mostly generated during downstroke where *C*_*Z*_ is positive. Mid-downstroke corresponds to the maximum lift production. During supination and early upstroke, the wings are still able to generate some weight support. Around mid-upstroke, vertical force becomes negative even though its amplitude is not particularly high.

**Table 3.** Force production and power of upstroke, downstroke and entire cycle (i.e. average between upstroke and downstroke).

	*C*_*Z*_	*C*_*T*_	*C*_*P*_	*C*_*Z*,b_	*C*_*D*,b_
whole cycle	0.466	0.115	0.266	0.266	0.151
downstroke	1.006	0.157	0.394	0.342	0.157
upstroke	−0.074	0.073	0.137	0.189	0.146
down-/upstroke ratio	—	2.15	2.88	1.81	1.08

On the other hand, figure 8*b* shows that thrust is mostly positive during both downstroke and upstroke. Furthermore, thrust has a greater peak during upstroke than during downstroke. However, the data in table 3 show that downstroke on average produces more thrust than upstroke.

 Figure 8*c* shows that the power consumption during both half wingbeats are significant. However, the power requirement is greater for downstroke, about twice as high as upstroke. This feature is similar to hovering, where downstroke power is nearly 2.8 times of upstroke power according to Song *et al.* [5], who studied the ruby-throated hummingbird. Using the equation $P=\frac{1}{2}C_{P}\rho U^{3}(2S)$ for the aerodynamic power, *P*, we have *P*=94.5 mW for the calliope hummingbird, and body mass-specific power 34 W kg^−1^. Thus, mass-specific power output of the hummingbird is within the range reported for larger bird species. For example, cockatiel power output ranges from 17 W kg^−1^ at 5 m s^−1^ to 47 W kg^−1^ at 14 m s^−1^, and dove power output ranges from 31 W kg^−1^ at 7 m s^−1^ to 54 W kg^−1^ at 17 m s^−1^ [31]. Compared with the hovering ruby-throated hummingbird at 55 W kg^−1^ [5], forward flight in the calliope hummingbird requires less power, which is expected since hovering generally is more energy-demanding than forward flight. On the other hand, forward flight at 8.3 m s^−1^ is not necessarily minimum power speed for the hummingbird, as the mechanical power output of birds can be described as a U-shaped curve function of the flight speed [31–33].

Using the present force coefficients and equation $F_{\mathrm{total}}=\frac{1}{2}(2C_{Z}+C_{Z,b})\rho U^{2}S$, we obtain the total vertical lift produced by the bird, which is around 94% of the bird weight. The bird body itself generates about 22.2% of body weight. This result will be discussed later. The thrust generated on the two wings together is 152% of the body drag. The imbalance of the vertical and horizontal forces could have been caused by several reasons: (i) the absence of camber in the wing model, (ii) error in digitization of the wing position, (iii) beak-feeder interaction as the bird was attempting to feed during the recording, (iv) interaction between the bird’s body and the wake of the feeder, and (v) an underestimate of drag coefficient for the body.

### 3.2 Force production mechanism

**Figure 9.**
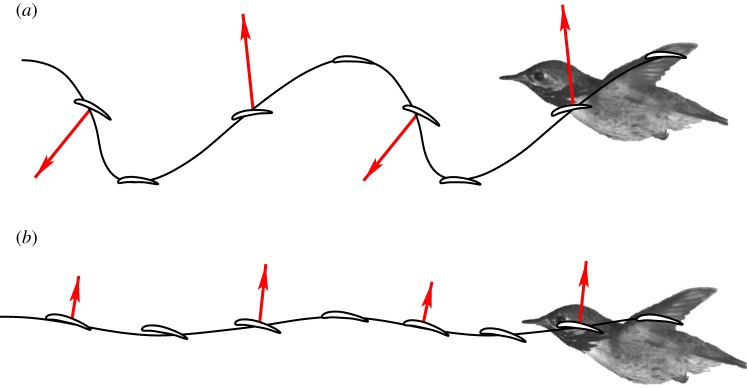
Time-dependent position of the distal chord (*a*) and proximal chord (*b*) in the global coordinate system with qualitative force production at mid-downstroke and mid-upstroke.

Overall force production of the hummingbird can be explained from the wing kinematics as viewed from the global coordinate system, i.e. the coordinate system fixed with the ambient air. Figure 9 shows the proximal and distal chord moving in the global coordinate system with their trajectories traced out. During downstroke, the angle of attack is positive for both the proximal chord and the distal chord. Therefore, both wing sections generate weight support. Since the leading edge of the distal section tilts downward, the aerodynamic lift has a forward component that leads to thrust generation during downstroke.

During upstroke, both wing sections move forward in air, even though the stroke plane angle is less than 90° and the wings move backward with respect to the body. Nevertheless, positive thrust is generated during this half cycle by the distal section. As shown in figure 9, the angle of attack of the distal chord is negative at upstroke, and the overall force on the section points downward and forward.

**Figure 10.**
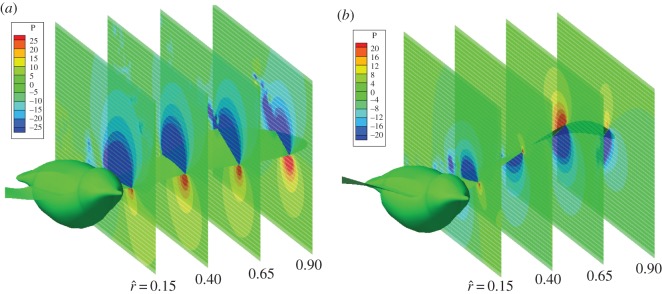
Pressure distribution (in Pascals) in the parasagittal planes at (*a*) mid-downstroke and (*b*) mid-upstroke.

 Figure 10 shows the pressure distribution within four selected vertical slices at mid-downstroke and mid-upstroke. It can be seen that the roles of the distal section and proximal section are different. For both downstroke and upstroke, the proximal wing has pressure surface on the ventral side and suction surface on the dorsal side. Thus, its main role is for vertical force generation. However, the distal wing flips its angle of attack between the two half cycles. Thus, positive (negative) pressure is distributed on the ventral (dorsal) side during downstroke, and the opposite is true during upstroke. This pressure differential leads to weight support during downstroke only, but thrust production during both downstroke and upstroke.

### 3.3 Vortex structures

Vortex structures, which are induced by the wing motion and dominate the wake, have been a focal point in the study of force production of flapping wings and fish fins. They can also be used to evaluate whether a bird adopts slow gait or fast gait [22]. As has been pointed out by previous researchers, at slow gait the trailing-edge vortices (TEVs) form rings after each downstroke, and the sequence would look like a series of smoke rings [34,35]; while at fast gait, the tip vortices (TVs) form undulating vortex tubes from the tip, and the TEVs form cylinders from the trailing edges, both being convected downstream [24,25,36].

In the current study of hummingbird flight, we used the iso-surface to show the vortex structures. The scalar quantity is defined as the maximum value of the imaginary part of the eigenvalue of the velocity gradient tensor ∇u, and it describes the strength of the local rotation of fluid [37]. It is found that the TVs are continuously shed from the wingtip, and the TEVs shed with the shape of separate cylinders. Such ladder-type vortex structures were also observed in the measurement of forward flight of swifts [25], which are closely related to hummingbirds, and like hummingbirds, do not flex their wings during flight. As pointed out by the authors [25], the ladder-type wake is formed by continuous shedding of the spanwise vortices from the wing surface and is different from an earlier speculation that for swifts and hummingbirds, a ladder-like wake would be generated by a distinct vortex in each of downstroke and upstroke [38]. Even though they both generate continuous ladder-like vortex structures, swifts do not forego weight support on upstroke like we report for hummingbirds.

**Figure 11.**
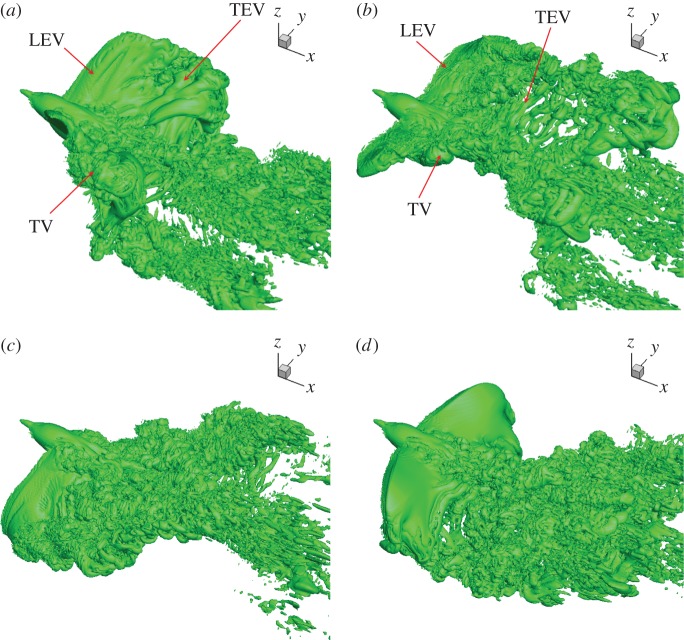
Vortex structures in the flow: (*a*) pronation, (*b*) mid-downstroke, (*c*) supination and (*d*) mid-upstroke. See the electronic supplementary material for an animation.

Several snapshots of the flow field are shown in figure 11. These snapshots show roughly the shape of the TVs that follow the trajectory of the wingtips. In addition, vortex shedding from the trailing edge is evident. Formation of the leading-edge vortices (LEVs) during both downstroke and upstroke is visible, and the LEVs are stable for both downstroke and upstroke. The behaviour of the LEVs has to do with both the instantaneous angle of attack and pitching rotation of the wing [39]. From figure 5*d*, the angle of attack of the distal section keeps a maximum value around 25° for a significant period of time, which would have caused LEV shedding and stall, if the wing simply translated without changing its pitch. However, the wings are also performing rapid pitching around their axes, as seen from variation of the chord angle plotted in figure 5*b*. That is, the chord angle magnitude decreases during downstroke after *t*/*T*>0.2, and quickly increases during upstroke before mid-upstroke. Such rotational motion has been known to maintain stability of LEVs and to enhance lift production of the wings [39].

### 3.4 Forces on the bird body

**Figure 12.**
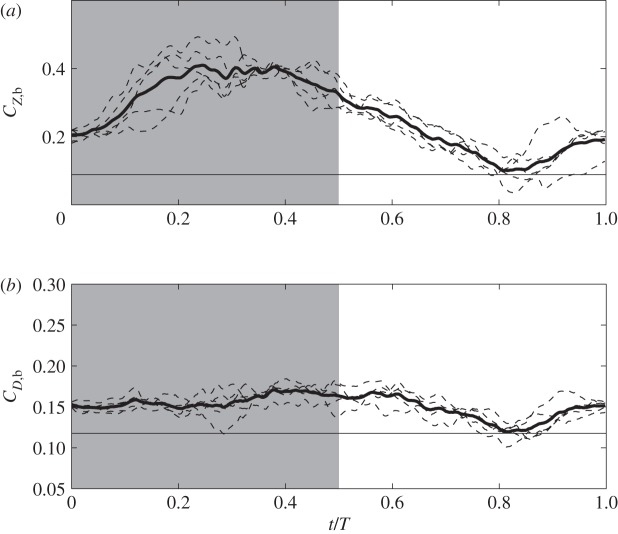
Instantaneous (thin dashed lines) and phase-averaged (thick line) lift (*a*) and drag (*b*) on the bird body. Thin solid lines denote data from the isolated body simulation.

Lift and drag on the bird body are affected by the orientation of the bird during flight. In general, the inclination angle of the body decreases with the increase of flight speed [12,23,27,40,41]. In the current study, the body angle of the hummingbird is *χ*_*b*_=12°, which is close to the angle of the rufous hummingbird at a speed of 8 m s^−1^, where *χ*_*b*_=11° [12]. Figure 12 shows both the instantaneous and phase-averaged data of the forces on the body. Downstroke-, upstroke- and cycle-averaged data are listed in table 3. These results show that the lift on the body provides 22.2% of the weight support. Furthermore, lift on the body during downstroke is 1.76 times of that during upstroke. In figure 12*a*, lift on the body oscillates significantly during a wingbeat cycle. On the other hand, drag on the body does not vary very much in a cycle and is nearly equal on average between downstroke and upstroke, which are reflected in figure 12*b* and table 3.

The high percentage of lift produced by the body and the oscillations of the body lift in a cycle may be attributed to aerodynamic interaction between the wings and the body that is assumed to be stationary in the current study. To verify this possibility, we also simulated separately the same flow around the isolated body without the wings attached. The shape and orientation of the body remain the same in the test.

**Figure 13.**
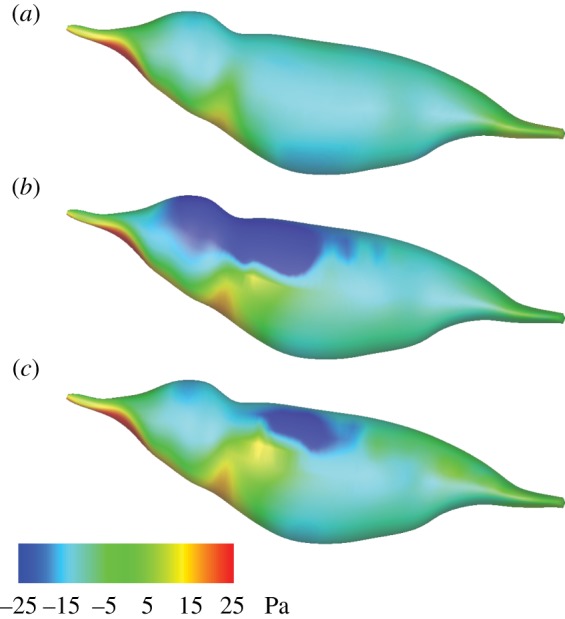
Pressure distribution on the body surface in Pascals: (*a*) isolated body simulation, (*b*) body with wings at mid-downstroke and (*c*) body with wings at mid-upstroke.

 Figure 13*a* shows the pressure distribution on the bird body from the isolated body simulation, which can be compared with the result from the full-body simulation shown in figure 13*b* and *c* for mid-downstroke and mid-upstroke, respectively. For the isolated body, even though a high-pressure zone is established below the body and near the head, the flow passes around the body in the absence of the wings and merges behind and above the body, where the pressure is partially recovered. As a result, the overall lift by the body is small. When the wings are present, the flow from below is prevented from passing around the body by the wings. Furthermore, the wing–wing interaction mechanism, similar to that proposed by Lehmann *et al.* [42], apparently has played a role here. That is, when the two wings are separating from one another from pronation to mid-downstroke, they create a low-pressure zone above the bird body, as shown in figure 13*b*, thus leading to a net upward force. This mechanism also explains why during upstroke, the low-pressure zone above the body, as plotted in figure 13*c*, is significantly smaller when compared with downstroke.

The present result shows that the bird body has significant contribution to the overall weight support. In comparison, previous experimental studies of insects and other birds indicated that the body lift is only a small portion of the animal weight, as shown in table 4. However, we point out that in those previous studies, the force was measured for the isolated animal body only, while in the current study, the wings are present and are in constant motion. For the isolated hummingbird body, we also observed low lift production. As shown in table 4, lift of the isolated hummingbird body is only 8% of the weight and is comparable with previous data for insects and also birds (e.g. 15–20% during flexed-wing bounds in zebra finch).

**Table 4.** Lift contribution from the body to weight support for different species. (Measurement for the insects was done on isolated bodies and for zebra finch was done using live birds with intact but folded wings and tails.)

taxa	speed (m s^−1^)	body angle *χ*	lift (% of weight)
hummingbird in flight	8.3	12°	22.2
(present study)			
hummingbird (body only)	8.3	12°	8.4
(present study)			
cockchafer beetle [43]	2–2.5	40°	3
Diptera [44]	2	10°	4
noctuid moth [45]	4	26°	10
bumblebee [15]	5	15°	8
zebra finch [46]	4–5	25°	15
zebra finch [47]	6–10	15°	20±5

### 3.5 Comparison of hummingbirds with other flying animals

**Figure 14.**
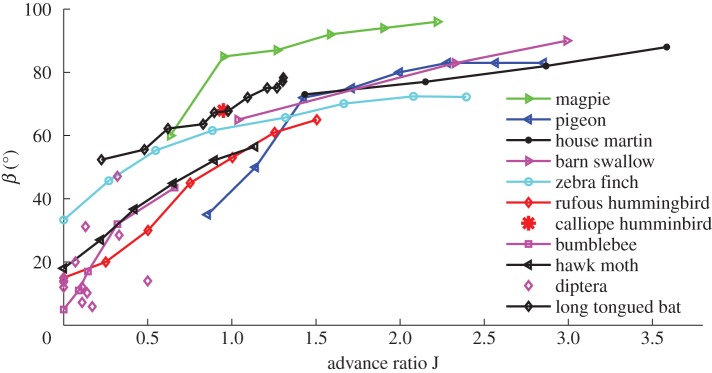
(*a*) Stroke plane angle versus advance ratio. Data are collected from the literature (*Diptera*: [48], bumblebee: [15,17], rufous hummingbird: [12], magpie, pigeon and zebra finch: [40,41], hawk moth: [49], barn swallow: [50], bat: [51]).

The advance ratio *J* and stroke plane angle *β* are two primary factors that affect the force production of flapping wings during forward flight. These two variables differ largely among animal species. Figure 14 shows a few species on the *β*−*J* map with the data directly collected or derived from various sources. It can be seen that hummingbirds largely fall within the range of the insects but also extend into the range of other birds. For all species, the stroke plane angle increases with the advance ratio, which is expected since at fast flight speed, the animals not only reduce the body angle for drag reduction, which would naturally cause the stroke plane angle to increase, but may also tilt the stroke plane further to enhance thrust generation.

**Figure 15.**
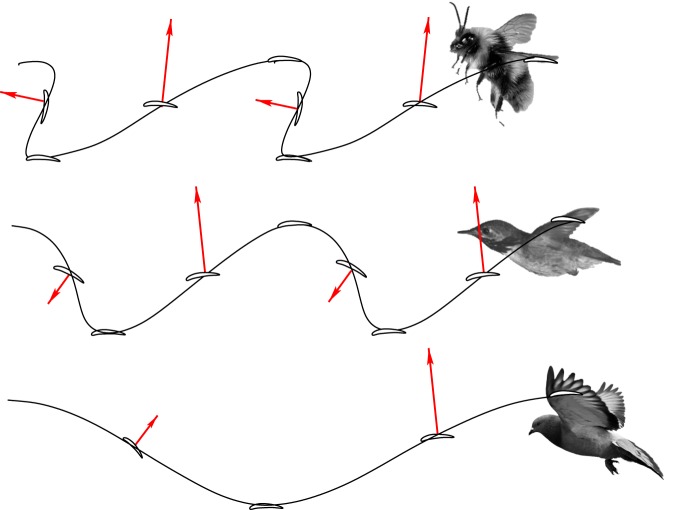
Wingtip trajectory and force production of bumblebee, hummingbird and pigeon.

For the small insects like bumblebees and fruit flies, the advance ratio is usually less than one [14]. For such slow flight, lift production is predominant over thrust production. Since the back-sweeping velocity of the distal wing exceeds the forward flight speed at upstroke [15,20,33], the wingtip trajectory traced out in the global coordinate system is highly backward skewed at upstroke, which is shown in figure 15 for a bumblebee at *J*=0.6. In this case, downstroke is mainly for lift production, while upstroke is mainly for thrust production. If the flight speed is further reduced, with a more skewed trajectory, upstroke may even produce lift as well. Overall, this strategy of using upstroke is also known as ‘backward-flick’ [18,23,31]. An exception is the fruit fly which is shown by a recent study that its upstroke uses a paddling mode to produce drag-based thrust [20].

For most birds, the advance ratio is significantly greater than one, and the stroke plane angle is close to 90°. Thus, the wingtip trajectory in the global coordinate system becomes much less backward-skewed and the wavelength-to-amplitude ratio becomes greater as shown in figure 15 for a pigeon. In this case, upstroke is not suitable for thrust generation. Instead, the wings are either feathered with little force produced [25,24], or swept on upstroke with some lift production (e.g. pigeon) [26,27]. On the other hand, a powerful downstroke is used to produce both lift and thrust.

For the hummingbird in the current study, the advance ratio is between that of insects and other birds. The wingtip trajectory is moderately skewed as shown in figure 15. Therefore, with proper angle of attack, the wings can still produce thrust during upstroke. However, since the overall force points downward, some lift has to be sacrificed. From this figure, it can be seen that thrust can be produced when the wing speed at upstroke is comparable to or possibly even lower than the flight speed. This thrust mechanism is analogous to a sail that moves against wind and thus is termed a ‘sail mode’ in this work. On the other hand, downstroke of the hummingbird is similar to that of big birds, as shown in figure 15, where both lift and thrust are generated.

It should be pointed out that at slow flight speeds, force production of the hummingbird still appears close to that of insects. As shown by Tobalske *et al.* [12], when *J* is below 0.7, the stroke plane angle of the hummingbird is small and the wingtip trajectory is also highly skewed like that of insects. Similarly, some insects can also perform fast flight at *J*>1, e.g. hawk moth, as seen from figure 14. It would be interesting to see whether their force production mechanism is similar to that described here for the hummingbird.

There is significant similarity between the hummingbird and bats, both being capable of hovering and forward flight at *J*=1 [52]. Recent flow visualization studies of bats have suggested that aerodynamic function of upstroke changes with forward flight speed [16]. At hovering and slow speeds, bat wings are inverted during upstroke to aid weight support; and at fast speeds, downstroke produces weight support and thrust, but upstroke may generate extra thrust at cost of negative lift. These features are similar to what we have reported for the hummingbird, which is interesting given that, unlike hummingbirds, bats flex their wings during upstroke.

It is also interesting to point out the similarities and differences between the hummingbird and the swift, as both of them keep their wings extended during the entire wingbeat cycle regardless of flight speeds. This is highly unusual for birds, as most birds flex the wings during upstroke [53]. Regardless of keeping the wings extended, the combination of spanwise twist and the stroke plane angle reported here for the hummingbird is a mechanism for permitting flight over a wide range of speed. For the swift, it is believed that wing twist is an important factor leading to optimal efficiency of flapping flight [54]. On the other hand, the swift’s flight style is dedicated to cruising flight, and it is not adept at hovering, and some swift species are even unable to accelerate into flight from a standing start without first dropping in altitude to gain velocity [55]. Therefore, one conclusion could be that the swift pattern of force development is optimized for more continuous weight support during cruising flight in a way that the closely related hummingbird does not achieve; while for the hummingbird, its evolutionary trajectory may have favoured the capacity for some thrust production at the expense of the more-constant weight support style in the swift [54]. This hypothesis merits testing in a broader phylogenetic context, as it is not appropriate to infer evolutionary trajectories from two-species comparisons [56].

## 4. Conclusion

Three-dimensional computer simulation has been performed to study aerodynamics of a hummingbird in fast forward flight and whose wing motion was captured by filming the bird flight in a wind tunnel. The finding places hummingbirds in an interesting position relative to insects and large birds. At a speed of 8.3 m s^−1^, the advance ratio of the hummingbird is between those of typical insects and large birds, and the simulation results show that the hummingbird uses a different strategy for lift and thrust production. In particular, its power downstroke generates both weight support and thrust just like other birds, but its upstroke further enhances thrust by setting the distal wings at a proper angle of attack with respect to the oncoming air, even though such thrust enhancement comes at cost of some negative lift at upstroke. These features are thus similar to those of bats flying at *J*=1. Ultimately, caution is necessary in interpreting our results as they emanate from the study of a single bird of one species. New studies in a phylogenetic context will be useful for understanding the evolutionary trajectories and selective pressures that drove the hummingbird flight style.

## Supplementary Material

ESM 1: Animation of wing motion of the hummingbird with the surface normal illustrated for the distal area.

## Supplementary Material

ESM2: Animation of the three-dimensional flow field visualized using the vorticity criterion described in the article.

## Data accessibility

Data available from the Dryad Digital Repository: http://dx.doi.org/10.5061/dryad.8ch1b.
