# Supplementary figures and images for: Three-dimensional simulation for fast forward flight of a calliope hummingbird
Source: R Soc Open Sci. 2016 Jun 8;3(6):160230. doi: 10.1098/rsos.160230 (PMC4929914; doi:10.1098/rsos.160230)

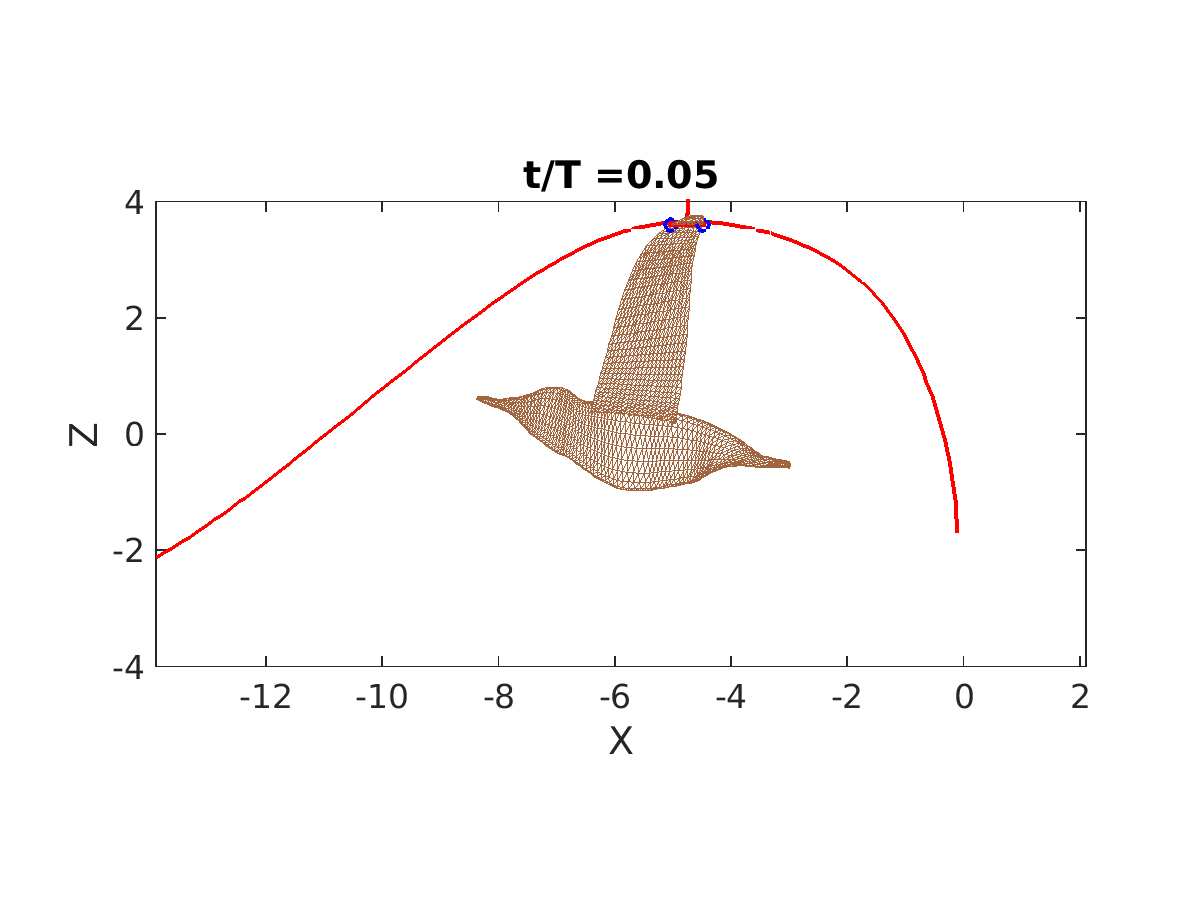

Supplement: ESM 1: Animation of wing motion of the hummingbird with the surface normal illustrated for the distal area. [file rsos160230supp1.gif]

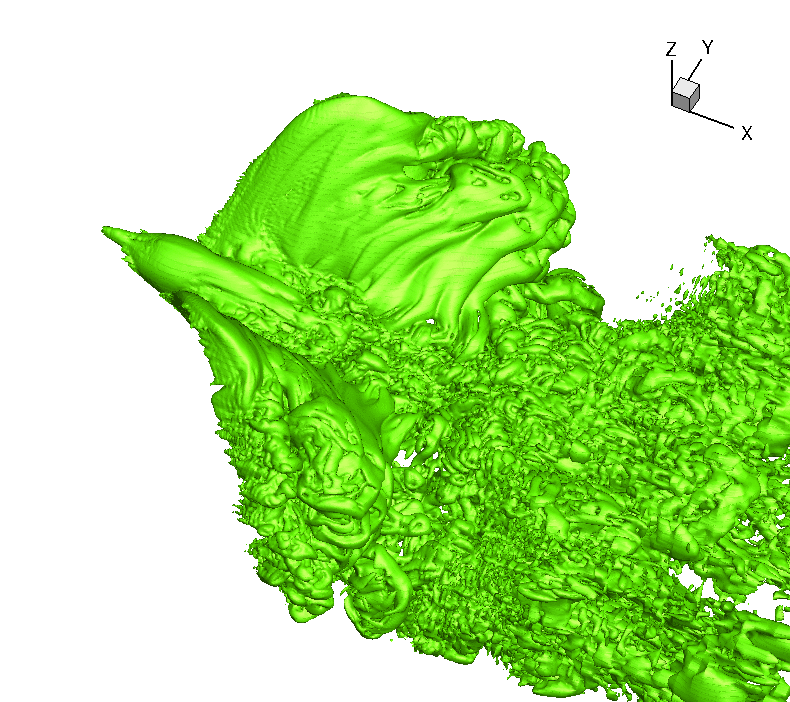

Supplement: ESM2: Animation of the three-dimensional flow field visualized using the vorticity criterion described in the article. [file rsos160230supp2.gif]
